# Modelling the Effectiveness of Gene‐Edited Salmon at Sea Lice Control and the Use of Refugia to Mitigate Counter‐Adaptation

**DOI:** 10.1111/eva.70166

**Published:** 2025-10-07

**Authors:** Andrew Coates, Nicholas A. Robinson, Tim Dempster, Ben L. Phillips

**Affiliations:** ^1^ School of Molecular and Life Sciences Curtin University Bentley Western Australia Australia; ^2^ Sustainable Aquaculture Laboratory—Temperate and Tropical (SALTT), Queenscliff Marine Science Centre Deakin University Burwood Victoria Australia; ^3^ Breeding and Genetics Nofima Ås Norway

**Keywords:** aquaculture, coho, metapopulation, model, refuge, resistance, sea lice

## Abstract

Advances in gene‐editing technologies offer opportunities to improve disease management in aquaculture. Gene‐editing applications for farmed Atlantic salmon ( *Salmo salar* ) include harnessing innate parasite resistance to protect against salmon lice ( *Lepeophtheirus salmonis* ). The potential for salmon lice to counter‐adapt to changes in the host should be considered. However, salmon farms are highly connected through louse transmission, and so it is important to gauge the impact of new technologies over large scales. Exploring the epidemiology and evolution of lice across a farm network is possible using metapopulation models. Here, we expand upon an eco‐evolutionary model to simulate the stocking of theoretical gene‐edited Atlantic salmon that rejected lice to a similar degree as the more resistant coho salmon ( *Oncorhynchus kisutch* ). Model outputs suggested that such louse resistance would be highly effective at controlling outbreaks and reducing the need for additional delousing treatments. Lice were controlled more efficiently when gene edits were prioritized at key farms in the louse dispersal network. In scenarios where gene edits selected for adaptive traits in the louse population, however, lice rapidly evolved counter‐resistance, leading to a significant reduction in treatment efficacy. When highly connected farms were left as refugia (not stocked with edited salmon), the rate of adaptation was slowed, thus extending the effectiveness of gene edits through time. The refuge effect was further enhanced if there were fitness trade‐offs to counter‐resistance in lice. We note that the long‐term benefits of the refugia approach—to individual farms and to the wider industry—must be balanced with the costs in the short term, especially for the refuge farms. Careful planning of how to distribute new technologies can maximize efficiency and help safeguard them against parasite evolution. Spatial eco‐evolutionary models are powerful tools for scenario testing that assist with decision making.

## 1 Introduction

Infectious diseases are a serious challenge to food production in aquaculture systems. The impact of disease outbreaks on farms can range from slower growth rates to mass die‐offs of stock (Blaylock and Bullard 2014; Lafferty et al. 2015; Sawabe et al. 2007). Transmission of parasites and pathogens between aquaculture sites (and to wild host populations) means a single outbreak can have widespread, cascading effects. Effective methods for disease prevention, containment, and control are thus critical components of a growing aquaculture sector (Barrett et al. 2020; Coates 2023; Murray and Peeler 2005).

Advances in gene‐editing technologies such as CRISPR Cas‐9 provide new opportunities to improve disease management in aquaculture (Robinson et al. 2023). Gene editing could introduce or enhance an innate disease resistance in livestock and prevent outbreaks from occurring in the first place (Barrett et al. 2020). Widespread stocking of disease‐resistant hosts could generate a ‘herd immunity’ effect, limiting infestations in more vulnerable populations. This may include suppressing parasite spillback into wild hosts.

When developing new control technologies, the potential for pest counter‐adaptation should be considered (Coates 2023). Given how new the technology is, there is limited research into whether gene‐edited livestock could elicit an evolutionary response in parasites. Parallels may be drawn with counter‐resistance to transgenic *Bt* crops, which are crops genetically modified to produce insecticidal *Bt* toxins derived from bacteria. At least 11 different insect species (across corn, soy and cotton hosts) have evolved resistance to *Bt* crops since their introduction (Tabashnik et al. 2023). The mechanisms behind the counter‐adaptations are varied across species (Heckel et al. 2007; Tabashnik et al. 2011). On the other hand, gene editing that alters the existing immune response of a host may constitute a more complex and nuanced suite of selection pressures, as opposed to the production of a specific toxin. The more genetic pathways that are involved, the more difficult it may be for parasites to counter‐adapt (Bishop et al. 2002; Kiyosawa 1982).

Farms can adopt integrated pest management practices aimed at mitigating the risk of counter‐adaptations arising (Rimbaud et al. 2018). For example, establishing ‘refuge’ sites can significantly slow the evolution of resistance to transgenic *Bt* crops (Crowder and Carrière 2009; Tabashnik and Carrière 2017). Refugia are designated areas that do not use a specific control strategy—in this instance, non‐genetically modified hosts are farmed in the refugia. Refugia maintain a pool of pests with susceptible genes, which then dilute the frequency of resistant genes in the wider metapopulation through gene flow. This strategy is especially effective if pests with resistant genotypes are selected against (due to fitness trade‐offs) at the refuge sites (Bateman et al. 2020; Kreitzman et al. 2018). The success of the refuge approach depends in part on the number, size and location of refugia in the wider host pool (Crowder and Carrière 2009; Onstad et al. 2013; Sisterson et al. 2005). Another approach for slowing counter‐adaptation is gene pyramiding, whereby multiple genes for resistance are combined into one host strain. This can be highly effective at maintaining the durability of resistant crops against pathogens (Mundt 2018; Rimbaud et al. 2018). When there is high pest gene flow between farm patches or farms, it is important to assess the success of approaches such as refugia and pyramiding at the metapopulation level. Metapopulation models are thus valuable tools for predicting the epidemiology and evolutionary dynamics of pests across farm networks, in response to heterogeneous selection pressures (Bateman et al. 2020; Kemper et al. 2013; MacKenzie and Bishop 2001; Onstad et al. 2013).

### 1.1 Gene Editing in Salmon

An excellent case for this is in Atlantic salmon ( *Salmo salar* ) aquaculture, an industry in which applications of CRISPR Cas‐9 for disease management are active (Robinson et al. 2024). Farmed salmon is at the cutting edge of technological developments for disease management (Barrett et al. 2020; Brakstad et al. 2019). The parasitic salmon louse ( *Lepeophtheirus salmonis* ) is the most significant disease for the salmon industry in the Atlantic, costing hundreds of millions of USD each year (Abolofia et al. 2017; Costello 2006). Strategies for lice control are costly and can come with other problems including high stress, reduced growth and mortality of salmon (Overton et al. 2019; Sviland Walde et al. 2021; Walde et al. 2022), the risk of pollution by toxic compounds (McRae et al. 2021; Moe et al. 2019), and the rapid evolution of pesticide resistance in lice (Aaen et al. 2015; Fjørtoft et al. 2021).

Harnessing the innate louse resistance of salmon through gene editing is an enticing alternative to existing strategies. Genetic variation in the immune responses—and, in turn, susceptibility—to louse infestations exists within the Atlantic salmon population (Gjerde et al. 2011; Gjerde and Saltkjelvik 2009; Holm et al. 2015; Kolstad et al. 2005).

There are also significant differences between salmon species that researchers can look to for inspiration. The Pacific coho salmon, *Oncorhynchus kisutch* , is markedly more resistant to lice than Atlantic salmon (Braden et al. 2020). Coho salmon mount a heightened inflammatory and hyperplastic response to *L. salmonis* that results in lice being encapsulated by rapid tissue growth, and subsequently killed by an influx of granulocytic immune cells (Braden et al. 2015, 2023; Johnson and Albright 1992). In laboratory studies, > 95% of lice are removed by coho salmon in the first weeks post‐infection (Braden et al. 2023; Fast et al. 2002; Johnson and Albright 1992). CRISPR Cas‐9 could be used to up‐ or down‐regulate any genes in Atlantic salmon that are homologous to the genes in coho underlying these effective immune defenses (Robinson et al. 2023; Salisbury et al. 2024). The ultimate goal might be to produce and distribute a commercially viable, gene‐edited Atlantic salmon that rejects lice to a similar degree as coho salmon.

### 1.2 Metapopulation Effects

Salmon farms are highly ‘connected’ to one another through the transmission of lice in their free‐living, larval stages. In Norway, each aquaculture site is a node in a vast web of larval dispersal routes many kilometres long (Samsing et al. 2019). Controlling for lice at one site can thus have ripple effects across the network (Coates et al. 2023). By the same token, the connectivity of sites facilitates louse gene flow, and so has a direct influence on evolutionary processes in the parasite (Besnier et al. 2014; Coates et al. 2023; Kaur et al. 2017). Metapopulation models are thus an excellent tool for the salmon industry to explore how management practices at individual farms can influence the epidemiology and evolution of lice at a regional or national scale.

In this study, we expand upon the metapopulation model described in Coates et al. (2022) to explore the effects of a theoretical gene‐edited, louse‐resistant salmon on the population dynamics of *L. salmonis* in Norwegian aquaculture. Our simulations focus on how spatial heterogeneity in management (especially refugia) can be used to disrupt louse epidemiology and evolution. First, we assess the overall success of the gene‐edited salmon at controlling lice in the absence of any counter‐resistance in lice. This includes simulations exploring how to maximise metapopulation‐level louse control through the spatial distribution of edited fish. Next, we introduce multi‐locus counter‐resistance in lice into the model. We run scenarios testing how effective refugia and pyramiding strategies are at slowing louse adaptation, and we assess their costs and benefits to farms through time. The effect of genetic factors—the number of genes under selection and the presence of fitness trade‐offs to resistance—is also examined in separate scenarios.

## 2 Methods

### 2.1 Model Description

We expanded upon the metapopulation model described in Coates et al. (2022, 2023). The model is a deterministic, stage‐structured matrix model that tracks the number of lice on farms over discrete weekly time‐steps, *t*.

Lice are grouped according to genotype, *g*, and life stage, *b*. There are four life stages in the life cycle: ‘larva’, ‘chalimus’, ‘pre‐adult’ and ‘adult’; $b=\left\{L,C,P,A\right\}$, $l=\left\{L,C,P,A\right\}$, respectively. For one louse genotype and one farm site, the number of lice in each stage, *n*_*l*_, is calculated from one time‐step to the next with the matrix multiplication:

(1) $$N_{t+1,g}=S_{g}\cdot N_{t,g}=\left[\begin{array}{cccc} 0 & 0 & 0 & fu_{g}\\ \mathrm{dv} & \left(1-\delta \right)\mu_{C} & 0 & 0\\ 0 & \delta \mu_{C} & \left(1-\delta \right)\mu_{P} & 0\\ 0 & 0 & \delta \mu_{P} & \mu_{A} \end{array}\right]\cdot {\left[\begin{array}{c} n_{L}\\ n_{C}\\ n_{P}\\ n_{A} \end{array}\right]}_{t,g}$$

Within the transition matrix **S** are parameters describing louse life history: *δ*_*l*_ is the development rate (of stage *l*), *μ*_*l*_ is the background survival rate, *d* is the probability of larval self‐recruitment, *v* is the attachment rate of larvae, *f* is fecundity of adults, and *u*_*g*_ is the Hardy–Weinberg proportion of offspring expected to be genotype *g*. The parameters are described in more detail in Table 1.

**TABLE 1:** Summary of model parameters.

Indices	Description	Value
*t*	Time‐step	Represents 1 week
*l*	Life stage	Lice categorised as Larva, Chalimus, Pre‐adult or Adult: $l\in \left\{L,C,P,A\right\}$
*g*	Genotype	Lice categorised as SS, RS or RR: $g\in \left\{\mathrm{SS},\mathrm{RS},\mathrm{RR}\right\}$
*i*	Farm	Lice categorised by farm location (with metapopulation 537 farms in metapopulation): $i\in \left\{1,2\ldots 537\right\}$
Life cycle parameters		
*T*_*ti*_	Temperature (°C) at farm *i* and time‐step *t*	Average temperature over 5‐week periods Data from barentswatch.no
*δ*_*T*_	Proportion of lice developing to next life stage per week	Calculated according to temperature, *T*_*ti*_ Daily transition rate (from Hamre et al. 2019; coefficients averaged across sexes): $\delta_{\mathrm{day}}=0.000581T^{2}+0.0094805T+0.0047395$ Weekly transition rate (aggregated chalimus I & II, and pre‐adult I and II): $\delta =1-{\left(1-0.5\delta_{\mathrm{day}}\right)}^{7}$
*f*_*T*_	Average number of larvae produced per adult per week Assuming two egg strings per female and a 50:50 adult sex ratio, there is an average of one egg string per adult (Coates et al. 2022)	Calculated according to temperature *T*_*ti*_ Number of eggs per egg string (from Johnsen et al. 2021): $N_{\text{eggs}}=e^{\left\{5.6-0.43\times \frac{T}{10}-0.78\times {\left[\ln\left(\frac{T}{10}\right)\right]}^{2}\right\}}$ Days between clutches (from Johnsen et al. 2021): $D_{\text{hatch}}=0.25\times \left[\frac{5}{4.85e-4\times T^{2}+8.667e-3\times T+3.75e-3}\right]$ Number larvae per adult per week: $f=\left(N_{\text{eggs}}/D_{\text{hatch}}\right)\times 7$
*μ*_*l*_	Proportion of life stage *l* surviving per week (after background mortality)	Weekly survival rate: $\mu_{C}=0.986$ $\mu_{P}=0.838$ $\mu_{A}=0.838$ Estimated from daily mortality data (Stien et al. 2005)
*d*_*ji*_	Proportion of larvae dispersing from farm *j* to farm *i*	Values taken from particle‐tracking model outputs (Samsing et al. 2017) for each unique combination of *j* and *i*
*v*	Proportion of incoming larvae that attach to host	v=0.05
Treatment and resistance parameters		
*a*_*μ*_	Relative proportion of chalimus survival rate on salmon with gene‐edit *a* (multiplied to *μ*_*C*_)	$a_{\mu }=0.632$
*b*_*μ*_	Relative proportion of chalimus survival rate on salmon with gene‐edit *b* (multiplied to *μ*_*C*_)	$b_{\mu }=0.632$
*z*	Proportion survival after mechanical delousing	z=0.3
*α*_*R*_	Increase in chalimus survival (addition to *a*_*μ*_) for each R allele	$\alpha_{R}=0.15$
*α*_*T*_	Increase in chalimus survival (addition to *b*_*μ*_) for each T allele	$\alpha_{T}=0.15$

### 2.2 Model Correction and Testing

We have noticed an inaccuracy in the previous model calculations described in Coates et al. (2022, 2023). The parameter for weekly louse development rate, *δ*, was calculated in those studies directly from the equation provided in Hamre et al. (2019, table 1). This equation captures the temperature‐dependent development of lice through four life stages on a host before becoming an adult (chalimus I, chalimus II, pre‐adult and pre‐adult II). In our simplified matrix model, these stages are condensed into just two (chalimus and pre‐adult). Using the original *δ* value on the condensed life cycle means that lice transition through the chalimus and pre‐adult stages too rapidly. We have amended this by multiplying the daily transition rate by 0.5 (Table 1). This is because it will take double the time for lice to transition through one of the two aggregated stages in our model than it would to transition through one of the four stages (chalimus I, chalimus II, etc.) previously described. Although a relatively simple adjustment, the predicted louse development fits well with predictions from a more complex population model with daily (instead of weekly) time steps and with the chalimi and pre‐adults separated into four stages (Figure S1). Under the stage‐structured model, chalimi require 2 weeks to transition to the adult stage. Under the coldest simulation conditions (3°C), 1% of chalimi become adults in two time steps, compared to 50% under the warmest conditions (17°C), which falls within observed development rates (Hamre et al. 2019; Stien et al. 2005).

We ran the simulation for azamethiphos given in Coates et al. (2022) again, this time with the adjusted calculation for *δ*. The slower development rate translated to a slower rate of adaptation in the louse metapopulation (Figure S2). The increase in the frequency of the resistant allele lagged in the updated simulation compared to the original by three to 4 years. Apart from this delay, however, the spatial and temporal patterns in louse adaptation, abundance, and treatment frequencies were almost identical to those described in Coates et al. (2022). The conclusions given in Coates et al. (2022, 2023) are thus still relevant under the corrected *δ* parameter, albeit occurring under a slightly longer time frame.

### 2.3 Louse Genotypes

Lice are grouped according to genotype, *g*, over two biallelic loci. One locus carries the alleles R or S; the other carries the alleles T or U. Diploid lice thus have nine possible genotypes: $g=\left\{\text{RRTT},\text{RSTT},\text{SSTT},\text{RRTU},\text{RSTU},\text{SSTU},\text{RRUU},\text{RSUU},\text{SSUU}\right\}$. The two loci are unlinked. In this study, the S and U alleles are the ‘wild‐type’ louse alleles, and R and T are the resistant alleles (here, R and T improve survival on gene‐edited salmon). The effects of R and T are described further below.

The proportions of each louse genotype produced during reproduction, *u*_*g*_, are calculated according to the Hardy–Weinberg principle. For example, the proportion of offspring produced with the RSTU genotype (*u*_*RSTU*_) is equal to 2*p*_*R*_ *q*_S_*2*p*_*T*_ *q*_*U*_, where *p*_*R*_, *q*_*S*_, *p*_*T*_ and *q*_*U*_ are the frequencies of each allele in the parent population.

Equation (1) is expanded into block matrix form to capture all nine genotypes at one farm:

(2) $$L_{t+1}=G\cdot L_{t}=\left[\begin{array}{ccccccccc} S_{\text{RRTT}} & F_{\text{RRTT}} & F_{\text{RRTT}} & F_{\text{RRTT}} & F_{\text{RRTT}} & F_{\text{RRTT}} & F_{\text{RRTT}} & F_{\text{RRTT}} & F_{\text{RRTT}}\\ F_{\text{RSTT}} & S_{\text{RSTT}} & F_{\text{RSTT}} & F_{\text{RSTT}} & F_{\text{RSTT}} & F_{\text{RSTT}} & F_{\text{RSTT}} & F_{\text{RSTT}} & F_{\text{RSTT}}\\ F_{\text{SSTT}} & F_{\text{SSTT}} & S_{\text{SSTT}} & F_{\text{SSTT}} & F_{\text{SSTT}} & F_{\text{SSTT}} & F_{\text{SSTT}} & F_{\text{SSTT}} & F_{\text{SSTT}}\\ F_{\text{RRTU}} & F_{\text{RRTU}} & F_{\text{RRTU}} & S_{\text{RRTU}} & F_{\text{RRTU}} & F_{\text{RRTU}} & F_{\text{RRTU}} & F_{\text{RRTU}} & F_{\text{RRTU}}\\ F_{\text{RSTU}} & F_{\text{RSTU}} & F_{\text{RSTU}} & F_{\text{RSTU}} & S_{\text{RSTU}} & F_{\text{RSTU}} & F_{\text{RSTU}} & F_{\text{RSTU}} & F_{\text{RSTU}}\\ F_{\text{SSTU}} & F_{\text{SSTU}} & F_{\text{SSTU}} & F_{\text{SSTU}} & F_{\text{SSTU}} & S_{\text{SSTU}} & F_{\text{SSTU}} & F_{\text{SSTU}} & F_{\text{SSTU}}\\ F_{\text{RRUU}} & F_{\text{RRUU}} & F_{\text{RRUU}} & F_{\text{RRUU}} & F_{\text{RRUU}} & F_{\text{RRUU}} & S_{\text{RRUU}} & F_{\text{RRUU}} & F_{\text{RRUU}}\\ F_{\text{RSUU}} & F_{\text{RSUU}} & F_{\text{RSUU}} & F_{\text{RSUU}} & F_{\text{RSUU}} & F_{\text{RSUU}} & F_{\text{RSUU}} & S_{\text{RSUU}} & F_{\text{RSUU}}\\ F_{\text{SSUU}} & F_{\text{SSUU}} & F_{\text{SSUU}} & F_{\text{SSUU}} & F_{\text{SSUU}} & F_{\text{SSUU}} & F_{\text{SSUU}} & F_{\text{SSUU}} & S_{\text{SSUU}} \end{array}\right]\cdot {\left[\begin{array}{c} N_{\text{RRTT}}\\ N_{\text{RSTT}}\\ N_{\text{SSTT}}\\ N_{\text{RRTU}}\\ N_{\text{RSTU}}\\ N_{\text{SSTU}}\\ N_{\text{RRUU}}\\ N_{\text{RSUU}}\\ N_{\text{SSUU}} \end{array}\right]}_{t}$$

where **F**_**g**_ resembles **S**_**g**_ as given in Equation (1), but with all parameters except *fu*_*g*_ replaced with zeros (different genotypes only interact when there is recombination during the reproduction phase). The value of *u*_*g*_ corresponds to the genotype, *g*, of **S**_**g**_ and **F**_**g**_.

### 2.4 Metapopulation Structure

The metapopulation in this study was comprised of 537 populations, representing farm sites throughout southern Norway (58.4°–66.4°N). The proportion of larvae transmitted from any one farm to another, *d*, was parameterised from Samsing et al. (2017), who used a hydrodynamic particle‐tracking model to predict the survival and dispersal of larvae between the 537 sites. This particle‐tracking model is a well‐established tool for predicting louse dispersal in Norway, and its outputs have been strongly correlated with observational data (Johnsen et al. 2021; Myksvoll et al. 2018; Sandvik et al. 2016, 2020).

Equation (2) is replicated across every farm site, *i*, to encompass the louse metapopulation, **P**:

(3) $$P_{t+1}=M\cdot P_{t}=\left[\begin{array}{cccc} G_{1} & C_{21} & \cdots & C_{i1}\\ C_{12} & G_{2} & \cdots & C_{i2}\\ \vdots & \vdots & \ddots & \vdots \\ C_{1i} & C_{21} & \cdots & G_{i} \end{array}\right]\cdot {\left[\begin{array}{c} L_{1}\\ L_{2}\\ \vdots \\ L_{i} \end{array}\right]}_{t}$$

where **M** is a 537 × 537 block matrix. On the leading diagonal of **M** is the per‐farm transition matrix **G**_i_ (as given in Equation (2)) which contains the unique value of *d*_*ii*_, the probability of larval self‐infection, for that farm. A farm, *i*, only receives lice from other farms, *j*, through transmission of larvae. The connectivity matrices **C**_ji_ thus resemble **G**_**i**_, but with all values replaced by zeros except *d*_*ji*_ *v*, which represents the probability of the larvae (of each genotype) produced at farm *j* dispersing to and infecting farm *i*.

Weekly temperatures for each farm over 2012–2020 were obtained from the Norwegian farm database at barentswatch.no. Temperatures were averaged across years and over 5‐week periods (Coates et al. 2022) to give the average temperature per time‐step at each farm, *T*_*ti*_. As louse development and reproduction rates are temperature‐dependent, *T*_*ti*_ was used to parameterise *δ* and *f* at each time‐step and farm, according to the equations modified from Hamre et al. (2019) and Johnsen et al. (2021) (see Table 1). The number of salmon on each farm was also estimated so that louse populations could be expressed in abundance (individuals per host). This was calculated from the maximum allowed biomass per farm (Samsing et al. 2017), divided by the mean weight of salmon at harvest (4.5 kg; Barrett et al. 2022).

### 2.5 Gene Edits

For this study, salmon received two separate gene edits, referred to here as edits *a* and *b*, which increase the mortality of attached chalimi. The baseline weekly survival of chalimus, given by *μ*_*C*_ in the transition matrix **S** (Equation (1); Table 1), is used to describe survival on unedited salmon. The gene edits *a* and *b* are assigned corresponding parameters *a*_*μ*_ and *b*_*μ*_, which take values between 0 and 1 (Table 1). The edits further reduce chalimus survival by multiplying *μ*_*C*_ by the parameters *a*_*μ*_ and *b*_*μ*_. In the absence of counter‐resistance in lice, the survival of chalimus on salmon carrying both gene edits is thus given as: *a*_*μ*_ *b*_*μ*_ *μ*_*C*_. This calculation was used for lice with the ‘wild‐type’ SSUU genotype, which was assumed to be the most susceptible to the gene edits. The R allele conferred lice some resistance to the *a* edit, given by the parameter *α*_*R*_. For each R allele, the value of *α*_*R*_ was added to *a*_*μ*_. Likewise, the T allele conferred resistance to the *b* edit by adding *α*_*T*_ to *b*_*μ*_ for each T allele (Table 1). This assumed an additive genetic effect of the alleles at the two loci. The proportion survival of chalimi on gene‐edited salmon (with edits *a* and *b*) is calculated as:

(4) $$\text{chalimus survival}=\mu_{C}\left(a_{\mu }+\eta_{R}\alpha_{R}\right)\left(b_{\mu }+\eta_{T}\alpha_{T}\right)$$

where *η*_*R*_ and *η*_*T*_ are the number of *R* and *T* alleles in that genotype (each given the value of 0, 1 or 2). Values of *α* are capped so that adding them to *a*_*μ*_ and *b*_*μ*_ don't exceed values of 1 (i.e., *α*_*R*_ ≤ (1—*a*_*μ*_)/2).

### 2.6 Determining Parameter Values

We assigned parameter values that simulated salmon rejecting chalimus to a similar extent as coho salmon. To determine these values, we used data from Fast et al. (2002), in which louse infestations on both Atlantic and coho salmon were tracked over time in a laboratory setting. From 3 to 21 days post‐infection in that study, Atlantic salmon retained approximately 92% of their lice, compared to coho salmon, which retained 10% (Fast et al. 2002). The proportion of lice retained over this 18‐day period, *r*, was converted into the average proportion retained per week with *r* ^7/18^. The proportion weekly retention on Atlantic salmon was approximately 0.97, which was similar to our existing parameter for weekly chalimus survival: *μ*_*C*_ = 0.986. Weekly retention on coho was 0.4 (or 0.42 times that of lice on Atlantic salmon). Accordingly, we assigned *a*_*μ*_ *b*_*μ*_ = 0.4. This corresponded to *a*_*μ*_ and *b*_*μ*_ each individually having the value of 0.632 (Equation 4). To replicate the resistance observed in coho salmon, only the chalimus stage was removed by gene edits in this model—the later, motile stages were assumed to evade host defences.

We assigned both *α*_*R*_ and *α*_*T*_ the value 0.15 (Table 1). This value was chosen as it corresponds to a selection differential of approximately 1.5 for lice homozygous for resistance at a single locus, and of 2.2 for lice homozygous at both loci (Table 2). Selection differentials were calculated by dividing chalimus survival by the chalimus survival for the SSUU genotype. Based on the results of Coates et al. (2023), this is strong enough selection to produce a relevant metapopulation‐level response, whilst still being orders of magnitude smaller than the selection imposed on louse genotypes by chemical treatments (Coates et al. 2021).

**TABLE 2:** Proportion weekly chalimus survival on gene‐edited salmon for each louse genotype, from: (*x*_*μ*_ + *η*_*R*_ *α*_*R*_) (*y*_*μ*_ + *η*_*T*_ *α*_*T*_)*μ*_*C*_, given the parameters in Table 1. Counter‐resistant louse alleles (R and S) are given in bold.

Louse genotype			
R/S locus	T/U locus	Weekly chalimus survival	Selection differential
**RR**	**TT**	0.86	2.17
**R**S	**TT**	0.72	1.82
SS	**TT**	0.58	1.47
**RR**	**T**U	0.72	1.82
**R**S	**T**U	0.6	1.53
SS	**T**U	0.49	1.24
**RR**	UU	0.58	1.47
**R**S	UU	0.49	1.24
SS	UU	0.39	1.00
All louse genotypes on unedited salmon		0.986	

*Note:* The selection differential imposed on the genotype is relative to the SSUU genotype (i.e., dividing by 0.39).

These values also meant that the R and T alleles did not confer complete resistance to the gene edits. The proportion of chalimus survival for each genotype on gene‐edited salmon is given in Table 2. The proportion of survival of RRTT chalimi on gene‐edited salmon was 0.86, corresponding to a 13% reduction in survival compared to unedited Atlantic salmon (Table 2). This is similar to the rates of chalimus mortality observed on rainbow trout (Fast et al. 2002).

### 2.7 Mechanical Delousing

In addition to gene‐edited salmon, all farms in the model had access to mechanical delousing, which was deployed whenever adult louse abundance on a farm exceeded the maximum lice limits of 0.5 adult females per fish, or 0.2 adult females during weeks 16–21 (spring), which Norwegian farms are required to remain under (Sandvik et al. 2021). In the model, these limits corresponded to 1 adult per fish, assuming a 1:1 sex ratio (Coates et al. 2022), or 0.4 adults per fish during weeks 16–21. Abundances were calculated by dividing the number of lice by the estimated number of hosts at the farm (Coates et al. 2022). When a mechanical treatment was deployed, louse numbers were multiplied by the parameter *z*, representing the proportion survival from delousing. We set *z* = 0.3 for all genotypes and life stages (except larvae), based on Aldrin et al. (2023).

### 2.8 Running Simulations

The starting numbers of lice per farm were assigned according to actual louse abundances recorded for each farm in the first week of the year, averaged across 2015–2022 (from data available in barentswatch.no). Our simulations began with a 2‐year spin‐up period without any gene edits (all farms using only mechanical delousing), which allowed the metapopulation to settle into a regular seasonal cycle of infestations and treatments (Coates et al. 2023). After 2 years, gene‐edited salmon were simultaneously added to farms according to a set distribution strategy (discussed below). The simulation was run for another 15 years with the gene‐edited salmon.

Unless otherwise stated, louse populations at the start of all scenarios were comprised of 5% RSUU, 5% SSTU and 90% SSUU genotypes, corresponding to gene frequencies of 0.025 for both the R and T alleles. These gene frequencies are similar to the low frequency of the gene for azamethiphos resistance, observed in louse populations during the early stages of the pesticide's use (Fjørtoft et al. 2021; Kaur et al. 2016).

### 2.9 Scenarios

The distribution of gene‐edited salmon through the metapopulation was varied between simulations. In all scenarios, each farm was stocked with just one type of salmon (edited or unedited).

In assigning distributions of edited salmon across farms, we ranked farms according to a metric of outgoing ‘connectivity’, which represented the strength of larval dispersal between farm sites. Connectivity was calculated as the sum of all *d*_*ji*_ values for each farm (ignoring the probability of self‐infection, *d*_*ii*_), from the connectivity matrix produced by Samsing et al. (2017). This represents the total probability of larvae produced at a farm *i* dispersing to any other farm *j*. Farms with high outgoing connectivity have a high likelihood of transmission to other sites.

We simulated the following scenarios:

#### 2.9.1 No Gene Edits Added

This was run as a control simulation. All farms only used mechanical delousing for the duration of the simulation.

#### 2.9.2 No Louse Counter‐Resistance

To assess the effect of the gene edits without louse adaptation, simulations were run with only wild‐type SSUU lice. In this set of scenarios, gene‐edited salmon were stocked on:

- Random 25% of farms.
- Random 50% of farms.
- 25% of farms with highest outgoing connectivity.
- 50% of farms with highest outgoing connectivity.
- All farms.

#### 2.9.3 Without vs. With Refugia

This scenario included counter‐resistance in lice, conferred by the R and T alleles, as described above. In the simulation without refugia, all farms were stocked with gene‐edited salmon (with both *a* and *b* edits). In the refugia simulation, the 5% most connected farms by outgoing dispersal (those that transmit a large proportion of lice) were stocked with unedited salmon (i.e., maintained as refugia). The remaining farms were stocked with gene‐edited salmon.

#### 2.9.4 Fitness Trade‐Offs

We also explored the effect of refugia when there are fitness trade‐offs associated with resistance. In this scenario, the R and T alleles reduced chalimus survival on non‐edited salmon. To calculate survival on unedited salmon, the parameter *μ*_*C*_ was multiplied by (1 − *η*_*R*_0.025 − *η*_*T*_0.025), whereby 0.025 is subtracted from 1 for every copy of the R and T alleles for that genotype. For example, the relative survival of RRTT chalimus on unedited salmon is 0.9 times (1 − 0.05 − 0.05) that of SSUU survival. Gene edits in this scenario were distributed the same as in the ‘Refugia’ scenario. We focus here on a trade‐off simulating genotype incompatibility between host and parasite. Alternative trade‐offs were also explored, which gave similar results (see Supporting Information).

#### 2.9.5 Pyramiding vs. Separating Gene Edits

We compared simulations in which gene edits were pyramided in salmon (i.e., carrying both the *a* and *b* edits) or separated across salmon. In the pyramiding scenario, all farms were stocked with gene‐edited salmon. In the separated scenario, a random 50% of farms were stocked with salmon carrying only the *a* edit; the remaining farms were stocked with salmon carrying only the *b* edit. Counter‐resistance to the *a* and *b* edits was conferred by the R and T alleles in lice, respectively, as described above.

#### 2.9.6 Digenic vs. Monogenic Counter‐Resistance

All farms were stocked with gene‐edited salmon (with *a* and *b* edits pyramided). In the digenic scenario, counter‐resistance in lice was conferred by both the R and T alleles, as described above. This was compared to the monogenic scenario, where resistance to both *a* and *b* edits was conferred solely by the R allele. To match the values given in Table 2, proportion chalimus survival on gene‐edited salmon was parameterised as 0.39, 0.626 and 0.86, for SS, RS and RR genotypes, respectively (T and U alleles had no effect). At the beginning of the simulation, 5% of lice were heterozygous (RS), and the remaining lice had the SS genotype.

### 2.10 Outputs

When looking at the model outputs, we focused on the number of mechanical delousing treatments used through time in each scenario. We took this to be a representative measure of the success of gene‐edited salmon. Farms greatly benefit from a reduction in delousing frequency, due to the significant financial and welfare costs of treatments. Since maximum lice limits were the trigger for delousing in the model, treatment frequency was also a direct measure of louse infestations reaching levels deemed unacceptable in the legislation.

We assessed the yearly mechanical delousing frequency over the study area, and estimated the financial cost of treatments per farm under the different scenarios. For this, we estimated the cost of mechanical delousing as 0.38 NOK per kg of salmon (from Iversen et al. 2017). For simplicity, we kept this value constant, although it is expected to rise with inflation (Walde et al. 2023). The model also assumed a constant biomass for each farm site (from Samsing et al. 2017). This approach meant that the cost was scaled by the fact that larger farms are typically more expensive to treat.

In our results, we examined the frequency of R and T alleles in the metapopulation through time to gauge the rate of adaptation under different scenarios (and how it corresponds to changes in delousing frequency).

## 3 Results

### 3.1 No Gene Edits

**FIGURE 1:**
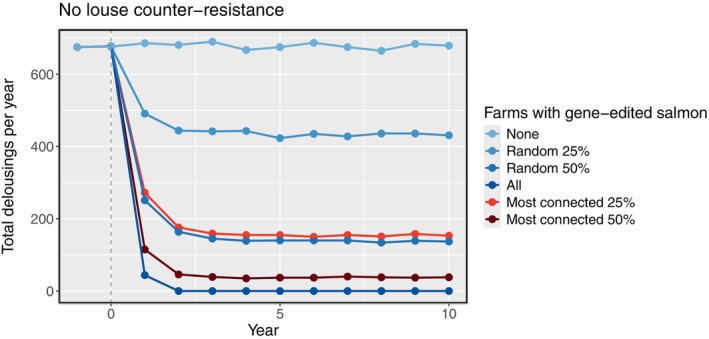
Total number of mechanical delousings deployed across all farms per year since stocking (vertical dashed line) of gene‐edited salmon, without any counter‐resistance in lice. The percentage of farms stocked with gene‐edited salmon ranged from 0% to 100%. Farms stocked with gene‐edited salmon were either determined randomly or according to larval connectivity values.

In the control simulation (no gene‐edited salmon and no louse counter‐resistance), approximately 680 (range 665–690) mechanical delousing treatments were performed per year across the study area (Figure 1).

### 3.2 No Louse Counter‐Resistance

When gene‐edited salmon were stocked at some farms, the frequency of mechanical treatments declined over the subsequent 2 years, before reaching a new equilibrium (Figure 1). Gene‐edited salmon had a much greater effect at controlling the metapopulation when they were used at farms with the strongest outgoing larval connections, compared to when distributed randomly. For example, when the gene edits were added to the same number of sites (25% of farms), the total mechanical treatments per year were reduced by either 40% or 77%, depending on if the sites were selected randomly or by highest connectivity, respectively (Figure 1). Without any counter‐resistance, stocking gene‐edited salmon at all farms was highly successful at controlling lice across the metapopulation. No mechanical treatments were required after 1 year of farming only gene‐edited salmon. After 7 years, lice had been effectively eradicated from the metapopulation.

### 3.3 Without vs. With Refugia

**FIGURE 2:**
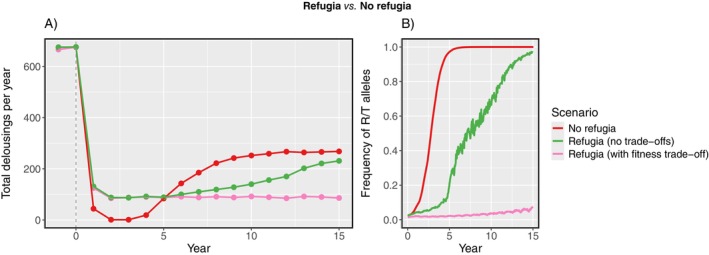
(A) Total number of mechanical delousings per year across farms, and (B) frequency of resistant R and T alleles in the louse metapopulation, since stocking of gene‐edited salmon. Either all farms were stocked with edited salmon (No refugia), or unedited salmon were left at the 5% most connected farms (Refugia). In refugia simulations, counter‐resistance in lice was modelled without fitness costs, and with a fitness trade‐off (reduced chalimus survival on unedited hosts).

With counter‐resistance present in the louse population, the frequency of mechanical treatments initially declined by ≥ 90% for the first 4 years after stocking gene‐edited salmon at all farms (i.e., without refugia). Delousing subsequently increased again as counter‐resistant alleles became widespread (Figure 2). The overall proportion of R and T alleles in the metapopulation rapidly increased to fixation over the first 10 years (Figure 2B). Over this time, treatment frequency increased to approximately 260 per year and then remained constant through the remaining years (Figure 2A).

With refugia (i.e., when the 5% most connected farms were left with unedited salmon), the overall delousing frequency was initially higher than without refugia (although there was nevertheless an 85% reduction from pre‐edit levels; Figure 2A). However, the rate at which the louse metapopulation adapted was much slower in the refugia simulation: it took 12 years for the gene frequency of the R and T alleles in lice to reach 90%, compared to only 4 years without refugia (Figure 2B). From years 6 to 15 of the simulations, fewer mechanical treatments were needed per year in the refugia scenario.

Over the entire simulation period, the cumulative number of mechanical treatments was 450 less in the simulation with refugia than without, equivalent to an accumulated 440 million NOK being saved overall. On a per‐farm basis, the refugia strategy reduced the total cost of treatments (over 15 years) for 54 (10%) of farm sites, compared to the scenario without refugia. Conversely, the refugia simulation increased treatment costs for 16 farms (3%), of which 13 were refugia sites, compared to when those sites were stocked with gene‐edited salmon. There was no difference in the total number of treatments for the remaining farms (including the other 13 refuge farms).

**FIGURE 3:**
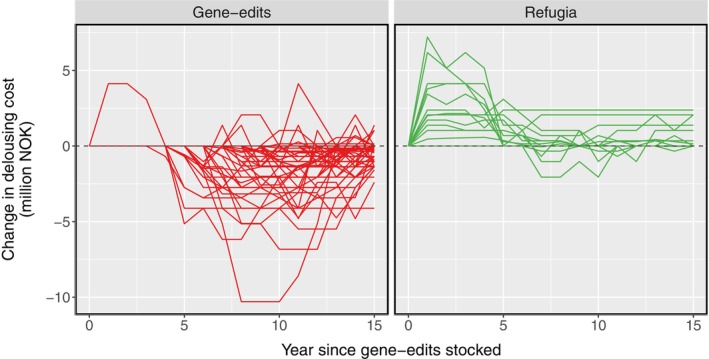
Difference in the yearly cost of mechanical delousing (million NOK) for each farm, with and without refugia used (negative values = refugia strategy decreased the treatment cost on a farm, positive = refugia increased cost). Left plot: Farms stocked with gene‐edited salmon in both simulations. Right plot: The 5% most connected farms that were refugia in the second scenario.

Figure 3 depicts the difference between the simulations with and without refugia in the treatment cost per year per farm. The lefthand plot depicts 95% of farms that received gene‐edited salmon in both simulations. The per year benefits from the refuge strategy (negative values in Figure 3) varied in scale (0.03–10.3 million NOK) and mostly occurred after 5 years. Conversely, of the 26 farms selected as refugia (the righthand plot), half experienced an increased yearly treatment cost in the refuge simulation (positive values), although this was most pronounced in the first 5 years (Figure 3).

**FIGURE 4:**
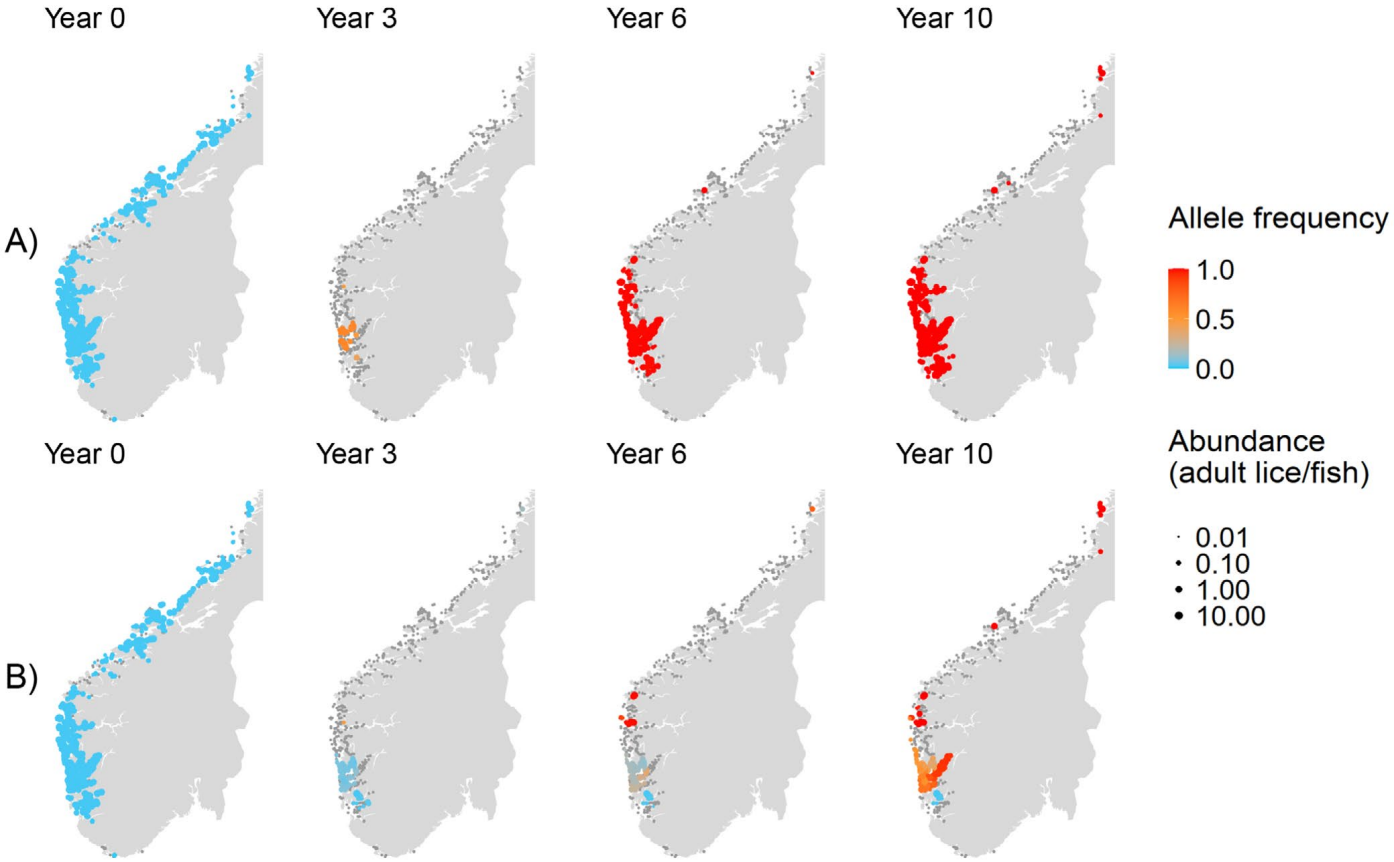
Louse metapopulation over 10 years following the introduction of gene‐edited salmon on farms, without refugia (A) and with the most‐connected 5% of farms as refugia (B). Each point represents a louse population at a farm, with colour indicating the frequency of the R and T alleles, size indicating adult abundance (lice per fish). Farms with abundances < 0.01 adults per fish are represented by grey points.

The spatial patterns associated with louse counter‐evolution are illustrated in Figure 4. The introduction of gene edits corresponded to an initial shrinking of the louse metapopulation as many farms extirpated most lice. Lice were maintained in the higher‐density region of farms in the south‐west, which became a hotspot of rapid evolution. Dispersal from this region led to farms being recolonised by resistant genotypes (Figure 4). When refugia were included, populations of susceptible lice persisted for much longer in the farm‐dense south‐west region (where the majority of refugia were located), which in turn slowed the rate of spread of counter‐resistant lice (Figure 4B).

### 3.4 Fitness Trade‐Offs

The effect of refugia was significantly enhanced when fitness trade‐offs to resistance were included. Over the 2‐year spin‐up period, the frequencies of the R and T alleles decreased from 2.5% to 1.4%. After gene‐edited salmon were stocked (with refugia), the frequency of these alleles increased incrementally, to only 7% after 15 years (Figure 2). Accordingly, the yearly frequency of mechanical treatments remained relatively constant (~90 per year; Figure 2). The louse metapopulation in this scenario was constrained to the south‐west of Norway (Production Zones 2 and 3).

### 3.5 Pyramiding vs. Separating Gene Edits

**FIGURE 5:**
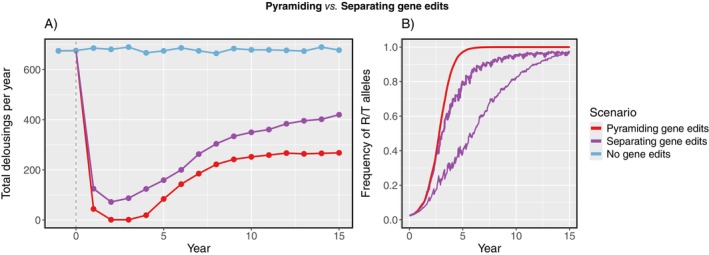
(A) Total number of mechanical delousings per year across farms, and (B) frequency of counter‐resistant R and T alleles (thick and thin lines, respectively) in the louse metapopulation, since stocking of gene‐edited salmon. Gene edits *a* and *b* were either combined in hosts (pyramiding) at all farms, or kept separate (*a* and *b* hosts were randomly distributed to an equal number of farms). The scenario without gene‐edited salmon is also included.

The metapopulation evolved more slowly when the *a* and *b* gene edits were separated across farms in different salmon strains than when the edits were pyramided (Figure 5). After 15 years, small frequencies of S and U alleles (~3%) were still present when edits were separated, rather than R and T becoming fixed as in the pyramiding simulation. This corresponded to mechanical delousing frequency increasing more gradually over the course of the separated simulation (Figure 5). At any given year, however, the total number of delousings was higher when edits were separated, compared to when pyramided in salmon. The spatial distribution of the edits amongst farms influenced the rate that each allele frequency increased: R increased more quickly than T in our simulation (Figure 5B). To test this, we ran additional simulations with different random distributions. Across those simulations the frequencies of R and T averaged out to be the same.

### 3.6 Digenic vs. Monogenic Counter‐Resistance

**FIGURE 6:**
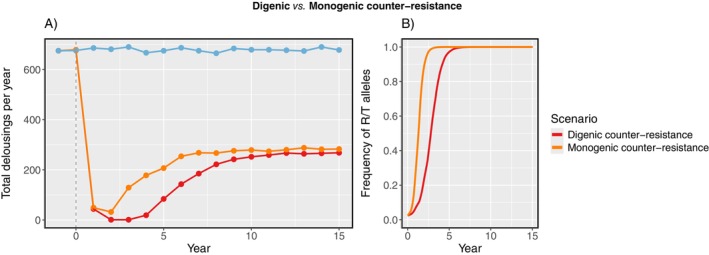
(A) Total number of mechanical delousings per year across farms, and (B) frequency of counter‐resistant R and T alleles in the louse metapopulation, since stocking of gene‐edited salmon at all farms. Counter‐resistance in lice was either digenic (conferred by R and T alleles) or monogenic (R allele only). The scenario without gene‐edited salmon is also included.

The louse metapopulation adapted more rapidly when counter‐resistance was conferred by a single allele than when it was digenic. Fixation of the resistant allele(s) occurred ~2.5 years sooner and the new metapopulation equilibrium (of 280 treatments per year) was reached ~4 years sooner with monogenic resistance (Figure 6).

## 4 Discussion

This study demonstrates how metapopulation models can provide insights into the epidemiology and evolution of salmon lice in salmon aquaculture under different management scenarios. Model simulations predicted our novel strain of gene‐edited salmon to be highly effective at controlling lice across southern Norway. The spatial distribution of edits across farms played a significant role in determining their success, especially when gene edits imposed selection for counter‐resistant traits in lice. Notably, we have shown that establishing a small group of highly connected farms as refugia (i.e., leaving these farms stocked with unedited fish) was effective at slowing the rate of louse adaptation, thus maintaining efficacy over a longer period. However, the long‐term benefits of refugia must be balanced with the short‐term costs, especially for the refuge farms.

### 4.1 No Louse Counter‐Resistance

Our simulations suggested that if farmed Atlantic salmon rejected early‐stage lice to a similar degree as coho salmon, then louse outbreaks could be significantly suppressed across a large‐scale salmon farm network. Our estimates of chalimus rejection rates were based on infection trials by Fast et al. (2002). Rejection rates observed in a more recent study (Sveen et al. 2025) are even higher (chalimus retention rate only 10% that of Atlantic salmon), suggesting that a coho level of louse resistance may be even more effective than simulated in this study.

Enhancing the immune response of Atlantic salmon to match that seen in other salmon species, via gene‐editing tools, thus holds the potential for effective, large‐scale management of lice. Current delousing methods (mechanical, thermal, freshwater and chemical removal) are costly to the industry, not only due to the operational costs involved, but also from the losses incurred through increased stress, injury and mortality of salmon post‐treatment (Overton et al. 2019; Walde et al. 2023). Frequent delousing also heightens the risk of lice developing resistance to the treatment (McEwan et al. 2016). Preventative measures such as lice skirts and submerged cages come with their own welfare considerations (Jónsdóttir et al. 2023; Warren‐Myers et al. 2022). Current selective breeding methodology may provide little group‐level protection (Ødegård et al. 2024). Gene‐edited salmon that innately remove lice bypass these issues and also reduce the need for additional delousing measures.

When stocking sites with gene‐edited salmon, the location of the farms stocked had as much of an impact on outputs as the number of farms stocked overall (Figure 1). Using edited salmon at key nodes in the farm connectivity network (i.e., stocking at farms with high probabilities of larval transmission, rather than randomly stocking the same number of farms) had a much greater effect at reducing outbreaks. This was because targeting lice at well‐connected populations significantly disrupted larval dispersal to surrounding sites. To achieve the same reduction in delousing rates, only half as many farms required gene‐edited salmon if stocking was done by connectivity, rather than randomly (Figure 1). Allocating resources in this way is important for new technologies, which are likely to have limited availability at first. The drawback to the above approach, however, is that clusters of farms with strong larval connections can also act as ‘hotspots’ of rapid evolution (as previously demonstrated by Coates et al. 2022). When gene edits imposed selection, connected sites facilitated the transmission of resistant genotypes to surrounding farms.

Another consideration when allocating resources is the proximity of farms to wild salmon migration routes and local trout populations. Regions where louse spillover is predicted to have an outsized impact on wild hosts would be good candidates for deploying strategies such as gene edits that act continuously through time to suppress lice levels.

### 4.2 Without vs. With Refugia

The refuge effect has become a valuable tool for slowing pest adaptation in agriculture, particularly to mitigate counter resistance to transgenic crops (Downes et al. 2010; Huang 2021; Tabashnik et al. 2023). Long‐term louse control was more effective when the 5% most connected farms were left as refugia—that is, stocked with unedited fish—compared to when gene edits were used at all farms, when edits imposed moderate selection on lice. Refuge farms supported susceptible louse populations, which then dispersed high frequencies of susceptible genotypes into the metapopulation, thus diluting the frequency of resistant alleles (Figure 4).

An important detail is that it took several years for the full benefits of refugia to be achieved in our simulations. It took 6 years for the yearly treatment frequency to be lower than in the no‐refuge scenario (Figure 2), but 8 years until the cumulative cost of treatments was lower, and 10 years until the cumulative number of treatments was lower. As seen in our first scenarios, gene‐edited salmon were very successful at controlling lice when stocked at the most connected farms (Figure 1). Omitting gene‐edited salmon from these key sites therefore significantly reduced the benefits over the first 5 years, even though gene edits were used in the remaining 95% of farms (Figures 2 and 3). For this approach to be successful at mitigating resistance, a long‐term plan must be adhered to over multiple production cycles, despite the initial drawbacks. As demonstrated by the model, resistance can still eventually become fixed in a metapopulation that contains refugia. The refuge effect is not necessarily a cure for resistance, but it can slow down the process of adaptation. This buys the industry more time to (a) detect resistance in the metapopulation, and (b) make informed decisions on additional actions to take (e.g., by swapping to alternative treatments in regions with high resistance).

Another drawback of the refugia approach is that half of the refuge farms required extra delousing treatments to keep lice under control—especially in the first few years—compared to when gene edits were used across all farms (Figure 3). When it comes to real‐world management decisions, it will be important to use models to ascertain whether the financial and welfare costs of additional treatments incurred at refuge sites are outweighed by the benefits to the wider farm community. To incentivise coordinating pest management across the industry, selected refuge farms could receive compensation: for example, they may be permitted to have higher louse limits to reduce delousing frequency (Kragesteen et al. 2019), or the cost of other control methods could be subsidised (Coates 2023). These decisions also need to consider welfare costs to salmon (e.g., whether allowing for higher louse limits is better than more frequent delousing treatments).

### 4.3 Fitness Trade‐Offs

The success of refugia at maintaining the efficacy of gene‐edited salmon was enhanced when fitness trade‐offs to resistance were included in the model (Figure 2). In our example scenario, louse genes that improved survival on gene‐edited hosts concomitantly reduced survival on non‐edited hosts. Adaptation to a novel host type can result in a parasite becoming maladapted to its original host in some cases; in others, the parasite can adapt without losing fitness on the original host. Either route can be taken within related groups of parasites (Khokhlova et al. 2022; Little et al. 2006). The ‘compatibility’ of both parasite and host genotype can determine infection success in some systems (Carius et al. 2001; Lambrechts et al. 2006; Salvaudon et al. 2007). Whether genotype‐by‐genotype interactions occur for *L. salmonis* —both within and between salmonid species—has not been studied. We focus here on a mismatch of parasite–host genotypes, but there are other ways that counter‐resistance to gene‐edited salmon could come with fitness trade‐offs. In other arthropod species, pesticide resistance comes with pleiotropic effects, including slower development rate, lower reproductive output and/or reduced longevity (Carriere et al. 1994; Foster et al. 2011; Gutiérrez et al. 2019; Higginson et al. 2005; Homem et al. 2020). Trade‐offs to development and fecundity in counter‐resistant lice were also simulated by our model and described in the Supporting Information (see Figure S3). We have previously shown using this model that metapopulation dynamics are affected by which life history parameter is under selection (Coates et al. 2023). Treatments that removed a proportion of lice from a cohort over multiple time steps (such as increasing the weekly mortality rate of chalimus) were highly effective at controlling outbreaks but also risked driving faster evolution as a result of more frequent selection.

### 4.4 Pyramiding vs. Separating Gene Edits

In crops, gene pyramiding—that is, stacking multiple resistance genes into one cultivar—is considered to be a highly durable strategy against pest counter‐adaptation. However, model demonstrations of this often assume an asexual pathogen, which accumulates counter‐resistant alleles through mutation (Mundt 2018; Rimbaud et al. 2018). In our simulations, by contrast, resistant alleles were already present at a low frequency in the louse metapopulation and were accumulated in offspring from sexual reproduction. Also, the durability of gene pyramids relies on resistant genes killing a very high percentage (> 95%) of susceptible pests—for diploid pests, this includes a very high mortality for heterozygotes (Carrière et al. 2015). In our model, gene edits had a more modest effect, as we wanted to replicate the mortality rates observed for lice on more resistant salmon species. As a result, lice evolved more rapidly when gene edits were combined in salmon than when they were distributed in a mosaic among farms (in the latter, each farm effectively acted as a refuge from one edit). Nevertheless, since louse control was maximized using pyramided gene edits, this was a better approach for louse management overall (Carrière et al. 2015).

### 4.5 Digenic vs. Monogenic Counter‐Resistance

As expected, it took longer for lice to adapt when resistance was determined by two unlinked loci, rather than when it was a monogenic trait. When resistance was digenic, each copy of the R and T alleles had a smaller individual effect, and so offspring had to receive twice as many resistant alleles from their parents to achieve the same fitness advantage as with monogenic resistance. Under Mendelian inheritance, the more loci that need to be accounted for, the lower the proportion of offspring with a particular genotype. In our simulations, the additional louse locus delayed adaptation by only a few years (Figure 6)—this is not likely to be much consolation for farms wanting to stock gene‐edited fish over several production cycles. If more genes (with smaller individual effects) were involved in counter‐resistance, then the rate of evolution would be slower (Palloix et al. 2009; Parlevliet 2002). Since the complexity of this model increases exponentially for every louse locus added, a quantitative trait model would be better suited to exploring metapopulation dynamics with resistance as a fully polygenic trait (e.g., Haridas and Tenhumberg 2018).

Our scenarios assumed that the two gene edits (*a*, *b*) in salmon corresponded to two adaptive genes (R, T) in lice. It is difficult to predict how many parasite genes would be required to overcome each host gene edit. Resistance to transgenic *Bt* crops is produced by a single corresponding gene in some pest species (Tabashnik et al. 1992; Yang et al. 2021), or by multiple genes in others (Janmaat et al. 2004; Taylor et al. 2021). The immune response of gene‐edited hosts may constitute a complex set of pressures (e.g., increased granulocyte proliferation, epithelial hyperplasia and/or suppression of louse immunomodulation) to which there might be no single gene solution in lice for counter‐adaptation. The durability of gene edits against louse counter‐evolution would be further enhanced if multiple loci for louse resistance are identified and edited in salmon.

Our simulations assumed both genes for counter resistance were present at low frequencies through the metapopulation, as with azamethiphos resistance (Kaur et al. 2017). If the R and T alleles originated in separate, localized areas, then multiple generations of lice would be required before the two alleles arrived in the same population. This would result in full resistance taking much longer to spread through the metapopulation. The rate of adaptation would also be affected if the resistant alleles had dominant or recessive effects (Coates et al. 2023).

### 4.6 Pathways to Counter‐Resistance

In our simulations, louse ‘counter resistance’ was given as an improvement in the survival rate of chalimi on gene‐edited hosts. The exact physiological or morphological traits coded by the R and T alleles were not needed for the model to function, allowing us to explore potential outcomes of the new technology before it is created. Variation in the immunomodulatory proteins produced by lice to evade and/or suppress host defenses (Fast et al. 2003, 2004; Midtbø et al. 2024) could be one avenue for such counter‐adaptation.

In our simulations, chalimus that were homozygous for both R and T alleles were not fully resistant to the gene edits, that is, they still had lower survival on edited salmon than on unedited fish. The survival rate of RRTT lice on gene‐edited hosts (Table 2) was similar to the survival of wild‐type lice on rainbow trout (Fast et al. 2002). Rainbow trout have an intermediate level of louse resistance, between coho and Atlantic salmon (Braden et al. 2020). Adjusting the fitness advantages conferred by R and T (i.e., the value of *α*_*R*_ and *α*_*T*_) would alter the selection differential imposed on lice, which would in turn influence the rate of louse counter‐evolution (Coates et al. 2023). There are alternative selection pathways which could be parameterised differently in the model. For example, lice could adapt to gene edits through an increase in chalimus development rate. Since gene‐edited salmon removed a subset of chalimus each time step, lice that spent less time in the chalimus stage (a higher *δ*_*C*_ value; Table 1) would have a higher proportion survival into the pre‐adult stage. There is already evidence of lice evolving a faster maturation rate in response to frequent delousing treatments (Mennerat et al. 2010, 2017). Temperature also had a subtle effect on the effectiveness of edits, with shorter chalimus durations at warmer temperatures, although we did not disentangle this from correlated geographical and seasonal effects. With warmer temperatures already increasing infestation levels (Hoddevik 2024), the effect of changing climate on louse dynamics would be an interesting use for this model in the future. Further modelling and simulation would be needed to explore such responses.

### 4.7 Model Limitations

This is the first demonstration of a metapopulation model for salmon lice that simulates both (1) a heterogeneous distribution of different control strategies across farms, and (2) selection imposed by treatments at multiple louse loci. The model can be customised to explore a range of management scenarios at the local or regional level. However, there are some fundamental assumptions and simplicities to the model that must be considered when interpreting the results.

First, just two types of control were simulated at any one time: gene‐edited salmon and mechanical delousing. This is in contrast to the many other technologies also used on farms, which vary in their efficacy and in their window of activity (Coates et al. 2021). The capacity to include them all in the model, and to deploy them in different combinations across the farm network, would add far more nuance to the model predictions. This will especially be the case if each strategy imposes selection on lice at different (or the same) loci. Chemical treatments are still used to varying degrees on Norwegian farms, despite known counter‐resistance in the louse population (Helgesen et al. 2024). Mechanical delousing is one of the most common delousing methods on Norwegian farms (Coates et al. 2021) and we used it here as the default treatment. Similar parameter values would suit simulations for thermal or freshwater delousing (Aldrin et al. 2023). Importantly, we assumed that lice do not adapt to these treatments, although there are concerns that non‐medicinal delousing could select for certain phenotypes (Coates et al. 2021; Ljungfeldt et al. 2017). The success of refugia or gene pyramiding to mitigate counter‐resistance becomes more complicated when the alternative treatments (e.g., mechanical delousing) also drive evolution. How various selection pressures interact at the metapopulation level is a compelling question for future modelling.

A second limitation is that our scenarios didn't explore temporal variation in management, and how it could be used to extend the longevity of treatments. For example, sites could alternate between stocking gene‐edited salmon and being refugia over different production cycles. Adding refugia to regions with escalating louse adaptation would relax selection for a period, and would be especially effective where there are fitness trade‐offs to counter‐resistance. Dynamic farm practices, more generally, were absent from our simulations: to focus on the role of larval connectivity, we assumed farms acted all in the same way through time (e.g., mechanical treatments applied only when infestations exceeded the legally mandated louse limits). Farms that treat for lice during the early stages of an infestation (well below the treatment threshold in our model) would help to prevent larger‐scale outbreaks that spill into surrounding farms.

Between‐farm differences in management practices could be incorporated as deterministic elements in the model (informed by real‐world farm data) or added as a stochastic element. Stochasticity could also be included to account for inherent randomness in population and evolutionary dynamics, including louse mutation.

### 4.8 Gene Editing Considerations

Gene‐editing research for Atlantic salmon is still in its infancy and laws do not currently permit the commercial production or release of gene‐edited fish in Norway and many other jurisdictions. Rigorous risk–benefit analyses should be conducted before any gene edits are implemented (Robinson et al. 2024). Important considerations include: the feasibility of disseminating edited stock at a commercial scale, the potential for side effects of gene edits on salmon health, the environmental impacts of escapees, and the securing of public and legal acceptance of gene‐edited foods (Robinson et al. 2024).

Here, in this study we simulated one possible set of scenarios involving gene‐edited salmon. There are other ways that knowledge about the genetic mechanisms in coho salmon could be implemented to improve louse resistance in Atlantic salmon (e.g., feed additives, selective breeding based on gene responses; Robinson et al. 2023). The simulations performed here are relevant to these other applications, provided that the result is a similar‐sized effect on host resistance. As the development and application of these technologies advance, the theoretical parameter values used here can gradually be replaced with values derived from empirical studies.

The encapsulation and mortality of chalimi through a coho‐like immune response is just one avenue by which Atlantic salmon could become more ‘louse resistant’. An alternative may be to alter the mucous composition of gene‐edited salmon so that they are less attractive to infective louse larvae in the first place (Robinson et al. 2023). Dedicated research is needed to assess whether there is genetic variation within the louse population in their ability to infect different host genotypes and how such variation may respond to selection pressures (Coates 2023; Lambrechts et al. 2006). Several studies into the genetic factors underlying pesticide resistance in lice exist, albeit after resistance has already become a widespread issue (Bakke et al. 2018; Igboeli et al. 2014; Kaur et al. 2017). The earlier the risks of counter‐resistance are identified for a new control technology (e.g., gene‐edited salmon), the sooner precautionary actions (e.g., the establishment of refugia) can be taken.

## 5 Conclusions

Our results indicate that enhancing louse resistance in Atlantic salmon to a level comparable with more resistant species (such as coho salmon) would provide substantial value in reducing delousing treatments and their associated costs. The spatial deployment of strategies across the farm network has a significant impact on their success at controlling louse outbreaks. However, it is also crucial to consider the influence of deployment on evolutionary dynamics. When the potential for louse adaptation was included in simulations, injudicious use of gene edits rapidly led to widespread counter‐resistance in lice. We have shown that establishing refugia at key sites in the transmission network can slow the rate of any adaptation, whilst still maintaining effectiveness at the metapopulation level. This strategy is especially powerful when there are trade‐offs to counter‐resistance in the parasite. As seen in our simulations, careful planning of how new technologies are allocated amongst farms can greatly improve the efficiency of louse control in the short and long terms. Spatial eco‐evolutionary models such as ours are powerful tools for scenario testing to assist with making these management decisions.

## Conflicts of Interest

The authors declare no conflicts of interest.

## Supporting information


**Data S1:** eva70166‐sup‐0001‐Supinfo.docx.

## Data Availability

The data and model code used in this article is available on GitHub at: https://github.com/coates‐a/salmonlice/releases/tag/v.2.0.
