# Supplementary material for: Modelling the Effectiveness of Gene‐Edited Salmon at Sea Lice Control and the Use of Refugia to Mitigate Counter‐Adaptation
Source: Evol Appl. 2025 Oct 7;18(10):e70166. doi: 10.1111/eva.70166 (PMC12504148; doi:10.1111/eva.70166)
Supplement: Supplementary file 1 — Data S1: eva70166‐sup‐0001‐Supinfo.docx. [file EVA-18-e70166-s001.docx]

**Updating louse development rate**

We compared the original and the updated louse transition rate, *δ*, using simple population models. We created three models that predicted the development of individual lice through their life cycle. In each model, a proportion of individuals, given by the parameter *δ*, transitioned to the next life stage with each time-step. The value of *δ* was determined by the equation given in Hamre et al., (2019):

$\delta_{day}=0.000581T^{2}+0.0094805T+0.0047395$,

where *δ_day_* is the daily transition rate and *T* is the temperature.

There was no mortality or fecundity. Models started with 100 chalimi (or 100 chalimus I in Model 3). The models were each run under three different temperature conditions: *T* = 8, 12 or 16°C.

**Model 1.** Lice were condensed into three life stages (chalimus, pre-adult, adult). Time-steps were weekly. The weekly transition rate, *δ*, was calculated from *δ_day_* with:

$\delta=1-\left( 1-\delta_{day} \right)^{7}$.

This was the method used in Coates et al., (2022). However, this approach predicts too-rapid louse development, because the Hamre et al., (2019) equation assumes five attached stages rather than three.

**Model 2.** As above, but *δ* was calculated from *δ_day_* with:

$\delta=1-\left( 1-{0.5\delta}_{day} \right)^{7}$.

This approach takes into account the aggregated life stages in our model. It assumes that one transition in our condensed life cycle (i.e., chalimus to pre-adult, or pre-adult to adult) takes twice as long as one transition in the complete life cycle (i.e., chalimus I to chalimus II, chalimus II to pre-adult I, etc.). This is the method used in the present study.

**Model 3**. In this model, lice were grouped into 5 stages (chalimus I, chalimus II, pre-adult I, pre-adult II, adult). Time-steps were daily. The value of *δ = δ_day_*. This is a more complex model that likely predicts louse development more accurately.

A comparison of the three models is given in Supplementary Figure 1.

*
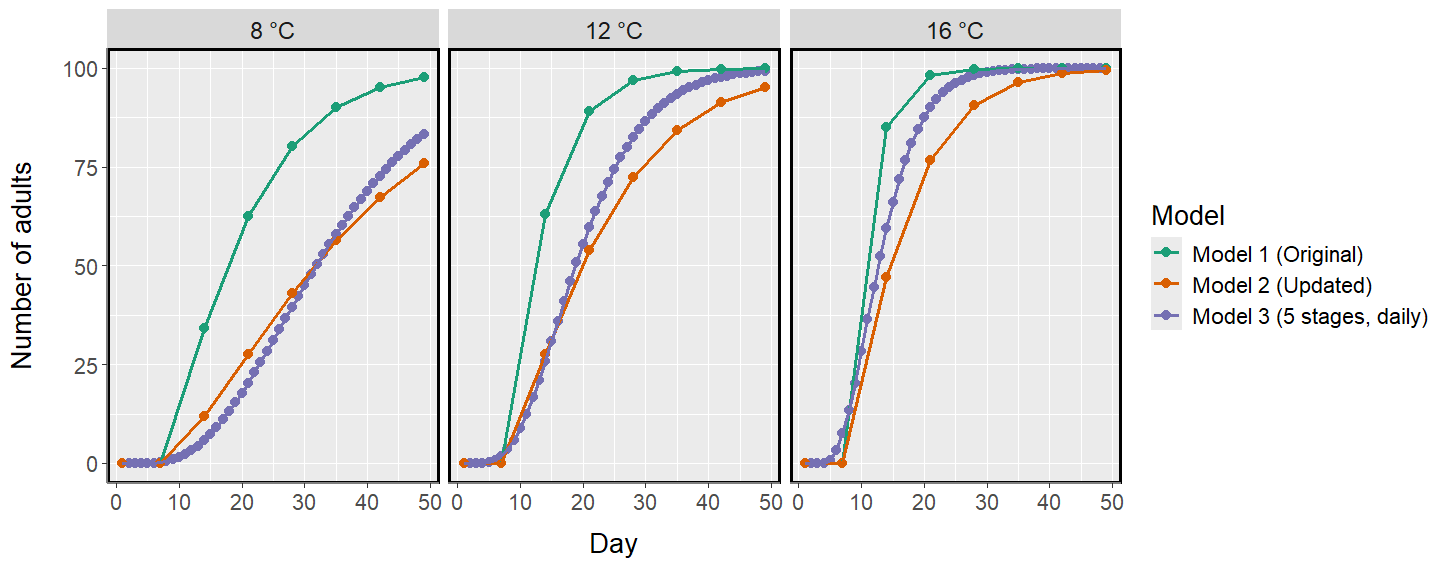
*

*Supplementary Figure 1.* Cumulative number of adult lice predicted per time-step under the three models (1 – 3, described above), at three temperatures.

The updated development rate of lice (Model 2; as used in the present study) predicted population dynamics that were closer to that predicted by Model 3. We posit that our adjusted version of the model likely reflects louse development more accurately. Although Model 3 is expected to be the closest representation of actual louse development, the added complexity makes it impractical to used in our full metapopulation model.

**Simulating the evolution of azamethiphos resistance**

We re-ran the simulation for azamethiphos resistance across Norwegian farms, as in Coates et al., (2022). We compared outputs parameterised with the original and the updated calculated for *δ* (the latter multiplying *δ_day_* by 0.5). The rates of adaptation in the metapopulation – given by the change in the frequency of the resistant R allele – are shown in Supplementary Figure 2.

*
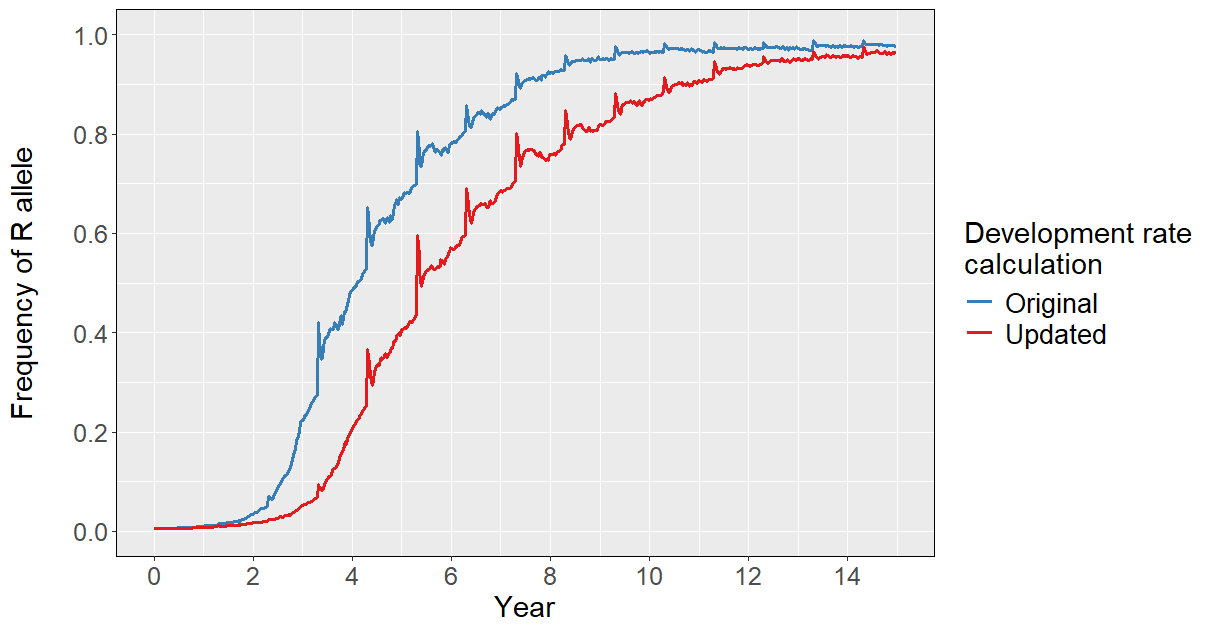

Supplementary Figure 2.* The frequency of the R allele (conferring resistance to azamethiphos) in the louse metapopulation through time. Simulations were run with the weekly louse transition rate, *δ*, calculated according to Coates et al., (2022; the ‘Original’ calculation) or per the present study (‘Updated’).

**Fitness trade-offs**

We explored the effect of alternative fitness trade-offs to louse counter-resistance. In addition to a trade-off to chalimus survival (as described in the manuscript), we also modelled scenarios where the R and T alleles imposed fitness costs to louse development rate or fecundity.

In the model, the life history parameters *δ* (weekly development rate for all attached stages) or *f* (weekly larval production of adults) were multiplied by (1 – *η_R_*0.025 – *η_T_*0.025), whereby 0.025 is subtracted from 1 for every copy of the R and T alleles for that genotype.

In the scenario with a trade-off to chalimus survival, the R and T alleles reduced survival on unedited salmon. Where counter-resistance incurred a trade-off to development or fecundity, we explored two sets of scenarios. In the first, trade-offs only occurred when lice infested unedited fish (simulating a mismatch in host-parasite genotypes). In the second set, the trade-offs occurred regardless of host type. The effects on delousing frequency are given in Supplementary Figure 3.


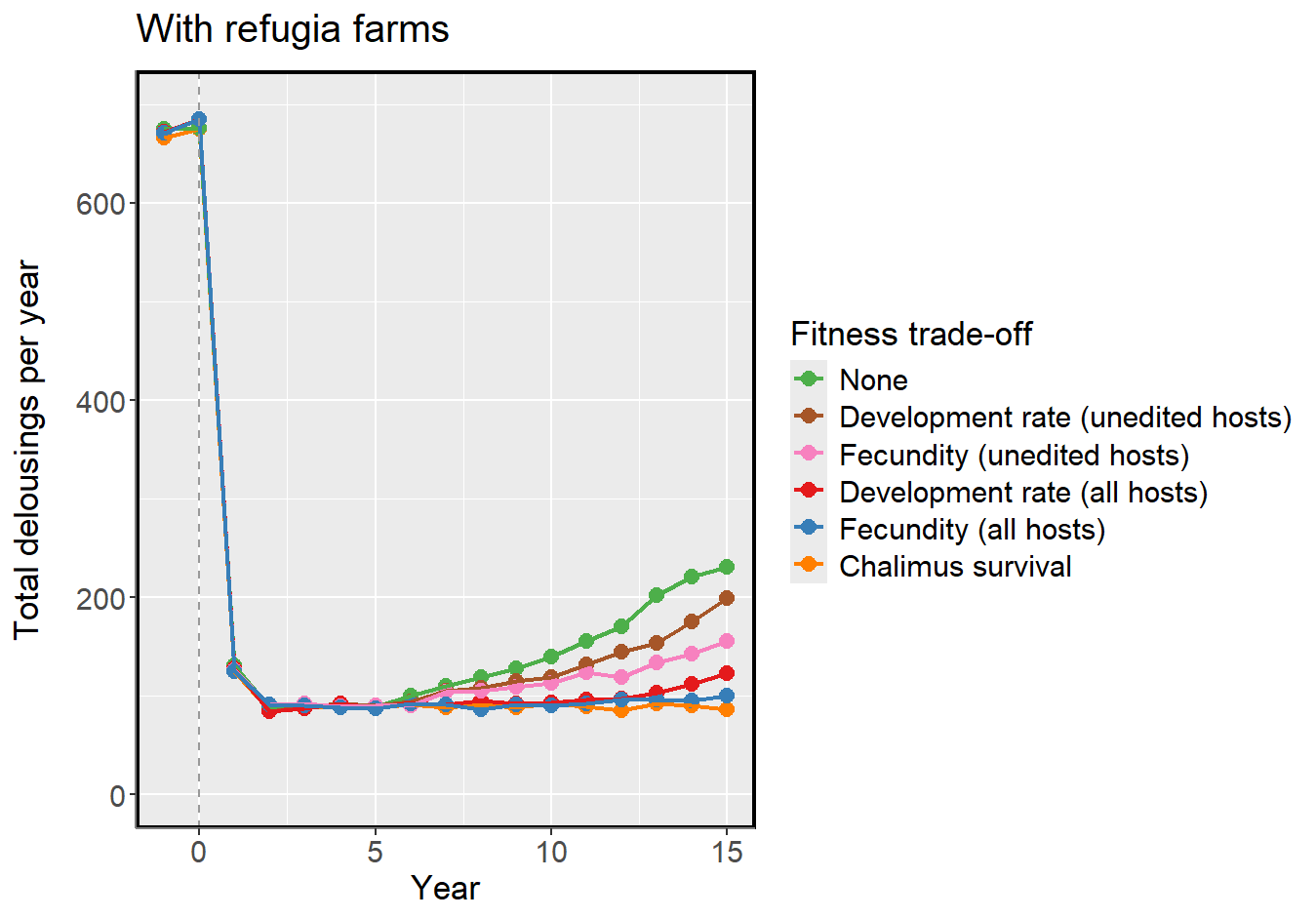


*Supplementary Figure 3*. Total number of mechanical delousings per year across farms since stocking of gene-edited salmon on 95% of farms (with the 5% most-connected farms left as refugia). The counter-resistant R and T alleles either had no fitness costs, or came with a trade-off to chalimus survival, development or fecundity. These trade-offs occurred on unedited hosts only, or all hosts.

When R and T imposed costs to development or fecundity, regardless of host genotype, the use of refugia had a similar effect to the scenario with a trade-off to survival. When those costs occurred only on unedited hosts, the benefits of refugia at slowing counter-adaptation was reduced. Lice levels were suppressed slightly more when resistant alleles reduced fecundity, compared to the same proportional reduction in development rate (as expected from Coates et al., 2023).
